# Resveratrol Inhibits Venezuelan Equine Encephalitis Virus Infection by Interfering with the AKT/GSK Pathway

**DOI:** 10.3390/plants10020346

**Published:** 2021-02-12

**Authors:** Caitlin W. Lehman, Kylene Kehn-Hall, Megha Aggarwal, Nicole R. Bracci, Han-Chi Pan, Lauren Panny, Robert A. Lamb, Shih-Chao Lin

**Affiliations:** 1 Department of Biomedical Sciences and Pathobiology, Virginia-Maryland College of Veterinary Medicine, Virginia Polytechnic Institute and State University, Blacksburg, VA 24061, USA; Woodsonc@vt.edu (C.W.L.); kkehnhall@vt.edu (K.K.-H.); nbracci@vt.edu (N.R.B.); laurenpanny@vt.edu (L.P.); 2 Department of Molecular Biosciences, Northwestern University, Evanston, IL 60208, USA; maggarwal@gsu.edu (M.A.); ralamb@northwestern.edu (R.A.L.); 3 Howard Hughes Medical Institute, Northwestern University, Evanston, IL 60208, USA; 4 National Center Animal Laboratory, National Applied Research Laboratories, Taipei 11599, Taiwan; hcpan@narlabs.org.tw; 5 Bachelor Degree Program in Marine Biotechnology, College of Life Sciences, National Taiwan Ocean University, 2 Pei-Ning Rd., Keelung 202301, Taiwan

**Keywords:** Venezuelan equine encephalitis virus, resveratrol, Akt, antiviral

## Abstract

The host proteins Protein Kinase B (AKT) and glycogen synthase kinase-3 (GSK-3) are associated with multiple neurodegenerative disorders. They are also important for the replication of Venezuelan equine encephalitis virus (VEEV), thereby making the AKT/GSK-3 pathway an attractive target for developing anti-VEEV therapeutics. Resveratrol, a natural phytochemical, has been shown to substantially inhibit the AKT pathway. Therefore, we attempted to explore whether it exerts any antiviral activity against VEEV. In this study, we utilized green fluorescent protein (GFP)- and luciferase-encoding recombinant VEEV to determine the cytotoxicity and antiviral efficacy via luciferase reporter assays, flow cytometry, and immunofluorescent assays. Our results indicate that resveratrol treatment is capable of inhibiting VEEV replication, resulting in increased viability of Vero and U87MG cells as well as reduced virion production and viral RNA contents within host cells for at least 48 h with a single treatment. Furthermore, the suppression of apoptotic signaling adaptors, caspase-3, caspase-7, and annexin V may also be implicated in resveratrol-mediated antiviral activity. We found that decreased phosphorylation of the AKT/GSK-3 pathway, mediated by resveratrol, can be triggered during the early stages of VEEV infection, suggesting that resveratrol disrupts the viral replication cycle and consequently promotes cell survival. Finally, molecular docking and dynamics simulation studies revealed that resveratrol can directly bind to VEEV glycoproteins, which may interfere with virus attachment and entry. In conclusion, our results suggest that resveratrol exerts inhibitory activity against VEEV infection and upon further modification could be a useful compound to study in neuroprotective research and veterinary sciences.

## 1. Introduction

As a highly threatening pathogen to livestock and humans, Venezuelan equine encephalitis virus (VEEV) has long been regarded as a neglected virus due to the low mortality rate in humans. However, the dissemination capability and morbidity induced by VEEV is devastating to equines, and occasionally, spillover to humans can occur [1]. VEEV is an enveloped, positive-sense, single-stranded RNA virus belonging to the *Alphavirus* genus of the *Togaviridae* family [2]. VEEV is harbored within *Culex* and *Aedes* mosquitoes and small mammals like rodents without causing symptoms, whereas infection with subtype IA/B and IC of VEEV (considered to be epizootic strains) might result in severe encephalitis and high mortality and morbidity rates in equids [3]. The incubation period for VEEV-infected horses is usually 1 to 5 days followed by febrile prodrome, tachycardia, and inappetence prior to neurological signs accompanied by diarrhea, dehydration, and severe weight loss. Infected equids could quickly succumb to the disease after the onset of encephalitis with or without symptomatic clinical signs. Permanent neurological disorders have been reported in recovered animals [4,5].

Despite the severity of VEEV, there is no United States Department of Agriculture (USDA)-approved vaccine and no effective anti-VEEV therapy available for equines, only supportive treatments [6]. Currently, the most widely used vaccine against VEEV is TC-83, a live attenuated strain generated from 83 passages of the Trinidad donkey (TrD) virulent strain in a guinea pig heart developed by the US Army Medical Research and Development Command (USAMRDC) [7]. Historically, the implementation of TC-83 vaccine in equids substantially controlled the outbreak from spreading northward during an epizootic/epidemic in Texas in 1971, where the death of over 1500 equines was reported [8]. However, there are some disadvantages with the use of TC-83 to immunize equids. Febrile responses and leukopenia along with depression and anorexia were observed in animals vaccinated with TC-83. The same study also observed the relatively low seroconversion rate in immunized subjects, rendering low to moderate titers of neutralizing antibodies against VEEV epizootic variants (IA/B and IC) [9]. Notably, it has been reported that VEEV can be isolated from mosquitoes in the same area where the horses were immunized with TC-83, enhancing the risk of VEEV dissemination [10]. As no anti-VEEV drug is available in the market, researchers are dedicated to the development of potent therapeutics while effective vaccines are still developing. In our previous studies, we identified that the U.S. Food and Drug Administration (FDA)-approved drug sorafenib as well as small-molecule inhibitors that target the host nuclear transport protein, importin, can reduce the infectivity of VEEV [11,12]. Unfortunately, these therapeutics are designed for humans, not equids or other mammals, and it may not be cost-effective to use these drugs on domestic animals. Hence, we intended to explore natural products for anti-VEEV activity and evaluate potential therapeutic effectiveness in vitro and in vivo.

Resveratrol (3,5,4′-trihydroxy-trans-stilbene), a natural phytoalexin, widely exists in plants such as grapes, mulberry, rhubarb, and peanuts as a form of polyphenol stilbene [13,14,15]. Resveratrol has been generally acknowledged to possess anti-oxidant, anti-aging, and anti-infection activities and is reported to be a neuroprotective agent [16,17]. To date, a broad range of viruses threatening non-human mammals have been documented to be suppressed by resveratrol, including African swine fever virus, pseudorabies virus, and influenza virus, all of which have caused an enormous economic loss in domestic animals [18,19,20]. Although resveratrol appears to broadly inhibit viruses, no report of resveratrol has been published regarding alphaviruses, such as VEEV. In recent years, the AKT/GSK pathway has drawn more attention in terms of alphavirus replication. Old World alphaviruses like Semliki Forest virus, chikungunya, and Ross River virus have been reported to utilize and activate the AKT pathway by phosphorylation, reprogramming the cellular metabolism and ultimately enhancing virulence and propagation [21,22,23]. As such, AKT appears to be a crucial target to be modulated in control of viral infection. The modulation of resveratrol to AKT has been repeatedly observed; resveratrol reduces the activation of the AKT pathway in various cell types, including neural cells, conferring its protective activity against infections or neural injuries [24,25,26,27,28]. However, the antiviral activity of resveratrol has not yet been evaluated against New World alphaviruses. Therefore, we aimed to assess the protection of resveratrol against VEEV along with other viruses, including Rift Valley fever virus, Zika virus, chikungunya virus, and Sindbis virus.

In this study, we investigated the antiviral potential of resveratrol using GFP- and luciferase-encoded VEEV. Time-of-addition analysis, apoptosis assays, and western blot analysis toward AKT-related host proteins were exploited for investigating possible mechanisms mediated by resveratrol acting on VEEV-infected cells. Additionally, the binding of resveratrol to VEEV glycoproteins was examined by molecular docking analysis and validated using other approaches.

## 2. Materials and Methods

### 2.1. Chemicals

Resveratrol used in this study was commercially provided by Sigma-Aldrich (St. Louis, MO, USA). Resveratrol was dissolved in dimethyl sulfoxide (DMSO) with a stock concentration of 100 mM and stored at −20 °C for further experiments. Prior to experiments, resveratrol was diluted to working concentrations from the range of 500 to as low as 1 µM in Dulbecco’s modified minimal essential medium (DMEM) supplemented with 2% fetal bovine serum (FBS).

### 2.2. Cell Viability Assays

The cell viability of Vero cells cultured with or without resveratrol in a 96-well plate was determined by measuring the amount of intracellular ATP [29] via the CellTiter-Glo^®^ Luminescent Cell Viability Assay (Promega^®^, Madison, WI, USA; Cat# G7570) according to the manufacturer’s protocol, which was also described in our previous report with the DTX880 Multimode Detector (Beckman Coulter, Brea, CA, USA). [12]. Cells were treated with the indicated concentration of resveratrol or DMSO solvent for 16 h prior to performing the CellTiter-Glo^®^ assay. The 50% cytotoxic concentration (CC50) values were calculated via non-linear regression fitted to the data by GraphPad Prism v7.0.

### 2.3. Luciferase Reporter Assays

Vero cells were seeded in 96-well plates overnight prior to infection with luciferase reporter virus, VEEV-TC83luc (a kind gift from Dr. Slobodan Paessler at the University of Texas Medical Branch) at an multiplicity of infection (MOI) of 0.1 for 1 h at 37 °C. Following removal of viral inoculum, viral replication in the presence (1 h pretreatment, co-treatment, and 1 h post-treatment) or absence of resveratrol (viral control) at 16 h post-infection (h.p.i.) was determined based on the intensity of luminescence using the Bright-Glo^TM^ Luciferase Assay System (Promega^®^ Madison WI USA. Cat# E2610) according to the manufacturer’s instruction and with DTX880 Multimode Detector (Beckman Coulter, Brea, CA, USA). The 50% effective concentration (EC50) values were calculated via non-linear regression fitted to the data by GraphPad Prism v7.0.

### 2.4. Plaque Assays

A monolayer of Vero cells was plated in 24-well plates (5 × 10^5^/well). The supernatants from viral infection experiments were collected and serially diluted 10-fold in DMEM. Diluted samples were added to the wells for 1 h at 37 °C with periodic rocking to evenly distribute the virus inoculum. Then, the overlay medium constituting a 1:1 ratio of 2× minimum essential medium (MEM) and 2% of agarose was added followed by 2-day incubation at 37 °C and in 5% CO_2_ before fixing and staining with 10% formalin and 0.2% crystal violet.

### 2.5. RNA Extraction and RT-qPCR

Intracellular total RNA along with viral RNA was isolated at 16 h.p.i using the RNeasy Mini Kit (Qiagen^®^, Hilden, Germany, Cat# 74106) according to the manufacturer’s instruction. RT-qPCR procedures were described previously [12] and performed using the UltraSense™ One-Step Quantitative RT-PCR System (Thermo Fisher Scientific, Waltham, MA, USA; Cat# 11732927), primer pair (forward TCTGACAAGACGTTCCCAATCA, reverse GAATAACTTCCCTCCGACCACA), and TaqMan probe (5′6-carboxyfluorescein-TGTTGGAAGGGAAGATAAACGGCTACGC-6-carboxy-N,N,N′,N′-tetramethylrhodamine-3′) on an Applied Biosystem StepOne Plus PCR system [30]. The absolute genomic copy number was calculated based on a standard curve established by known concentrations of VEEV-TC-83 RNA.

### 2.6. Western Blotting

Cell lysates were collected at indicated time points and extracted with lysis buffer, as described previously [12]. In brief, cell lysates with lysis buffer were loaded into InvitrogeN′s NuPAGE 4–12% Bis-Tris precast gels followed by transferring to a polyvinylidene fluoride (PVDF) membrane for further hybridization with the following primary antibodies: phospho-GSK-3α/GSK-3β at 1:2000 (Ser21/9, Cell Signaling, 9331), GSK-3β at 1:1000 (Santa Cruz, sc-71186), pan-Akt at 1:2000 (Cell Signaling, 4691), phospho-Akt (Thr308) at 1:2000 (Cell Signaling, 13038), LC3B at 1:1000 (Cell Signaling, 3868), and horseradish peroxidase (HRP)-conjugated actin (Abcam, ab49900). Following incubation of primary antibodies overnight at 4 °C, membranes were washed with 0.05% of Tween 20 in phosphate buffered saline (PBS) and incubated with corresponding secondary antibodies at room temperature for 1 h. Pierce ECL Western Blotting Substrate (Thermo Fisher Scientific, Cat# 32106) was applied for developing the luminescent signals on membranes.

### 2.7. Viral Attachment and Entry

For viral attachment, Vero cells in 12-well plates were pre-chilled at 4 °C for 1 h followed by infection of VEEV (MOI of 5) with or without resveratrol treatments at 4 °C for an additional 1 h prior to two washes with ice-cold PBS to remove the unattached viral particles. Next, 400 μL/well of complete medium was then added, and the plates were incubated at 37 °C for 2 h to allow the virus to be internalized. After incubation, the medium was removed and cell lysates were collected for viral RNA quantification. For viral entry, cells were pre-chilled for 1 h and subsequently infected with VEEV without resveratrol followed by washing out the inoculum with ice-cold PBS. Warmed complete medium containing resveratrol, along with no-resveratrol medium, was applied on cells, incubating at 37 °C for 2 h. Following incubation and gentle washes, cell lysates were then collected for further analysis at 16 h.p.i.

### 2.8. Molecular Docking and Dynamics

The cryogenic electron microscopy (cryo-EM) structure of VEEV structural proteins complex (3J0C) was retrieved from the Protein Data Bank (PDB) [31]. The mol2 file for the ligand resveratrol was obtained from the Zinc database [32]. Resveratrol was then docked in the VEEV E protein using Swiss-dock [33]. The top-scoring clusters were evaluated based on the binding free energy and manual visualization of the binding site in Chimera [34]. The molecular dynamics (MD) simulation for the docked molecule was performed using the Desmond module of Schrodinger [35].

### 2.9. Statistics

Unless noted otherwise, data were presented as means ± standard error of mean (SEM) and were compared using the Kruskal–Wallis test or one-way ANOVA for the determination of significance defined as *p*-value < 0.05.

## 3. Results

### 3.1. Evaluation of the Cytotoxicity and Antiviral Activity of Resveratrol against VEEV-TC-83 In Vitro

**Figure 1:**
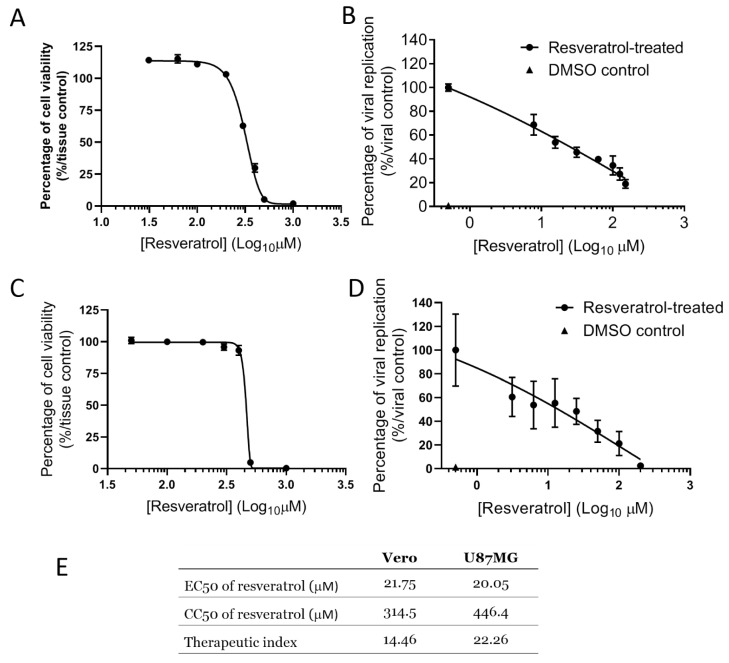
50% cytotoxic dose and effective dose (CC50 and EC50) of resveratrol against Venezuelan equine encephalitis virus (VEEV) in two distinct cell types. Vero (**A**,**B**) and U87MG (**C**,**D**) cells were treated with resveratrol at indicated concentrations (CC50) along with VEEV-TC-83 infection for 16 h at an MOI of 0.1 followed by measurement (EC50) of (**A**,**D**) cell viability and (**B**,**E**) replication of reporter VEEV-TC83luc quantified by cellular ATP and luciferase activity, respectively. The figures were plotted following the normalizing toward viral control groups. Viral control refers to no resveratrol-treated but VEEV-infected cells, while mock control was defined as cells treated with a solvent and without infection. (**E**) The CC50, EC50, and therapeutic index (TI) were estimated via non-linear regression with GraphPad Prism v7.0 to determine the 50% cell viability and effective dose. Data were presented as means ± SEM.

To assess the antiviral efficacy of resveratrol, we first established the 50% cytotoxic dose and effective dose (CC50 and EC50) of resveratrol in vitro. We directly applied resveratrol to Vero (monkey kidney) and U87MG (human glioma) cells at a wide range of concentrations (0 to 1000 μM) for CC50 determination. A luciferase reporter virus, VEEV-TC-83luc, was used to reflect the viral replication activity within host cells and thereby determine the EC50 by quantifying the luminescence. Following 16 h of resveratrol treatment, the CC50 was determined to be 314.5 (Vero) and 446.4 μM (U87MG) (Figure 1A,C). Resveratrol displayed an EC50 of 21.75 (Vero) and 20.05 μM (U87MG) (Figure 1B,D) against VEEV-TC-83luc. The therapeutic index (TI) of resveratrol on VEEV-infected Vero and U87MG cells was thereby found to be 14.46 and 22.26, respectively (Figure 1E). Our initial results indicate that resveratrol could potentially interrupt VEEV replication in multiple cell types without inducing significant cytotoxicity.

### 3.2. Resveratrol Suppressed the Propagation of VEEV in a Dose-Dependent Manner

**Figure 2:**
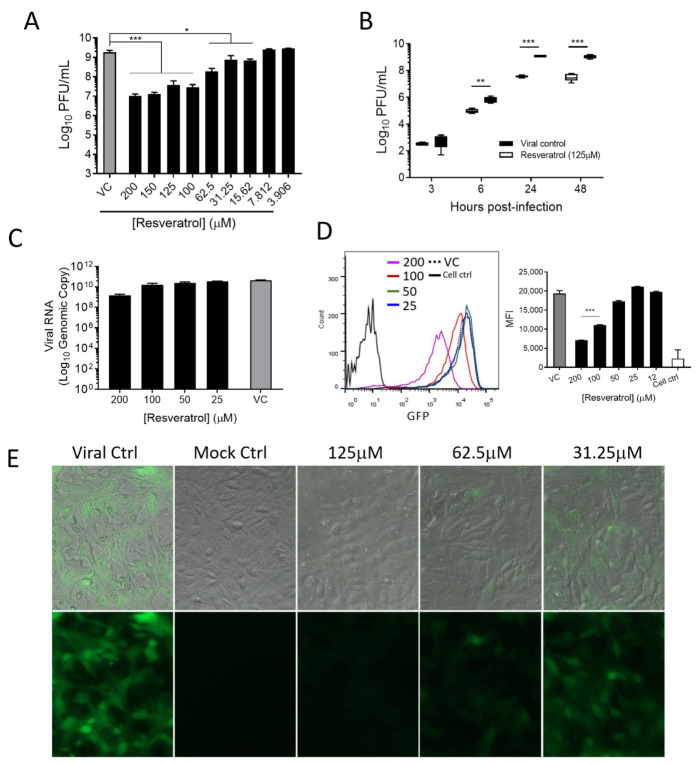
VEEV replication and propagation of infectious virions were assessed after resveratrol treatment. Supernatants from VEEV-TC-83-infected Vero cells (MOI = 0.1) were collected at (**A**) 16 hours post-infection (h.p.i.) with various concentrations of resveratrol and (**B**) at the indicated time points with 125 μM of resveratrol, followed by performing plaque assays to measure viral titers. (**C**) Total cellular RNA was isolated from infected cells after 16 h of VEEV-TC83 infection (MOI of 0.1) followed by conducting RT-qPCR to quantify the viral RNA. (**D**) GFP-encoded VEEV-TC83 was used to quantify and visualize the VEEV-infected cells at 16 h.p.i. by (**D**) flow cytometry and (**E**) fluorescent microscopy. MFI, median of fluorescence intensity; VC, viral control. Data were expressed as means ± S.E.M. * *p* < 0.05; ** *p* < 0.01; *** *p* < 0.001 compared to viral control (VC) by one-way ANOVA.

Upon establishing the antiviral activity of resveratrol, we sought to confirm whether the synthesis of infectious virions or viral genetic material was inhibited. To examine this, plaque assays and RT-qPCR were performed. We found that VEEV titers were proportionally reduced as the concentration of resveratrol increased. Infectious viral titers were stymied with ≥15.625 μM of resveratrol treatment (Figure 2A). Approximately, a 2-log decrease in viral titers was observed with 150 μM of resveratrol treatment, which was a dose that displayed no cytotoxicity. Furthermore, we treated Vero cells with a single dosage of 125 μM resveratrol followed by VEEV infection and measured viral titers at 3, 6, 24, and 48 h.p.i. Our data revealed that VEEV titers were suppressed by resveratrol as early as 6 h.p.i. and lasted for the entirety of the infection period (Figure 2B). Viral RNA production was also reduced at 16 h.p.i. with varying concentrations of resveratrol (Figure 2C). These results suggest that resveratrol displays a protective effect against VEEV replication and propagation.

Using a GFP-reporter VEEV-TC-83, we further examined the suppressive effect resveratrol treatment has against VEEV at 16 h.p.i. [36]. Fluorescent microscopy and flow cytometry found that VEEV replication, designated by GFP expression, dose-dependently decreased upon resveratrol treatment (Figure 2D,E). This mirrors the luciferase data. These data collected from multiple experimental approaches confirm that VEEV infection is continuously curtailed by resveratrol treatment in terms of infectious virion production and viral genomic contents.

### 3.3. Resveratrol Inhibited VEEV Replication over the Duration of Infection

**Figure 3:**
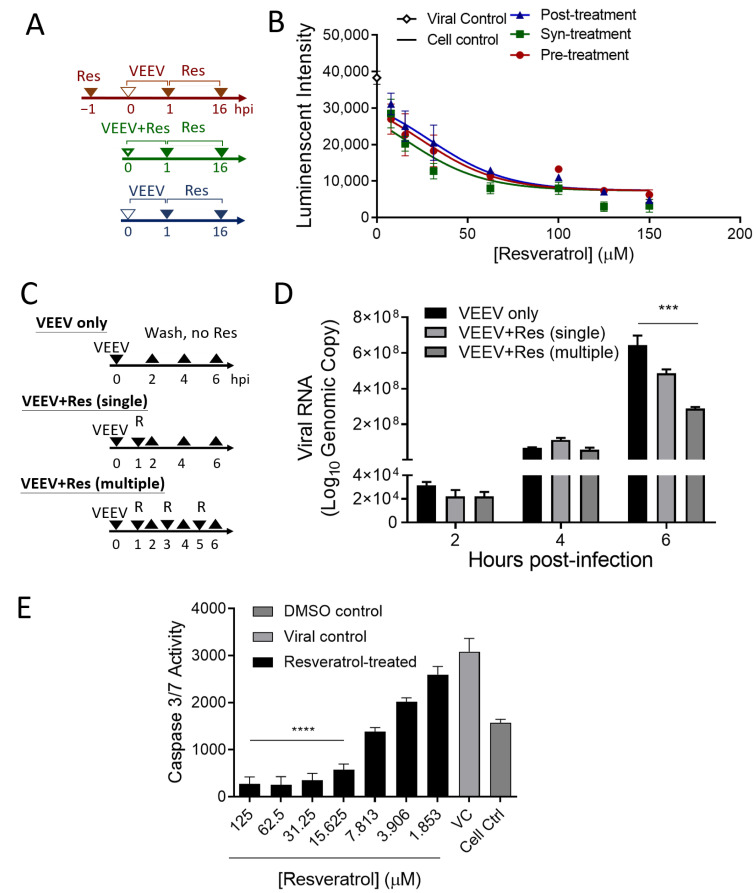
Time of addition of resveratrol marginally changed is antiviral effectiveness. (**A**,**C**) Schematic diagrams illustrating the experimental design for the time-of-addition experiments. (**B**) Luciferase activity and (**D**) viral RNA of VEEV-TC83luc at an MOI of 0.1 corresponding to the experimental designs were determined by BrightGlo^®^ assays and qPCR, respectively, at 16 h.p.i. (**E**) Apoptotic signaling activity of caspase-3 and caspase-7 in VEEV-infected Vero cells (MOI = 0.1) were measured with Promega’s Caspase-3/7-Glo^®^ assays at 16 h.p.i. *** *p* < 0.001; **** *p* < 0.0001 by one-way ANOVA. Data are represented as means ± S.E.M. VC, viral control.

To determine whether resveratrol has preventative and/or curative capabilities, a time-of-addition experiment was performed. Figure 3A depicts the various experimental methods used. Resveratrol was added to cells 1 h before (pre-treatment), after (post-treatment), or synchronously (syn-treatment) with the infection to address its timely effectiveness. The data indicate that the protection provided by resveratrol against VEEV is dominant regardless of the time of addition, evidenced with a similar trend in decreasing luciferase activity generated from active viral replication of VEEV-TC-83luc (Figure 3B). Notably, the treatment effect of resveratrol could still be achieved, even though the virus invaded host cells, which led us to perform the next experiment to test whether resveratrol can serve as an agent for post-exposure prophylaxis. As shown in Figure 3B, viral RNA content was measured at 2, 4, and 6 h.p.i. with a variable number of resveratrol doses (zero, one, or three doses) following infection. Our data indicate that the higher the number of doses, the greater the viral suppression, where we could observe significant viral replication suppression with multiple doses of resveratrol by 6 h.p.i. (Figure 3C,D and Supplementary Figure S1), suggesting that the anti-VEEV activity of resveratrol could occur extensively during the early phase of infection and can be implemented as post-exposure prophylaxis.

### 3.4. Resveratrol Reduced the Apoptosis Induced by VEEV Infection

We hypothesized that the repression of viral replication might be correlated with down-regulation of the cell death mechanism. To test this hypothesis, the activities of caspase-3 and caspase-7 and the percentage of apoptotic cells in resveratrol-treated cells were evaluated using the Caspase-3/7-Glo^®^ assay and flow cytometry, respectively. Caspase-3 and caspase-7 activities substantially reduced upon resveratrol treatments in a dose-dependent manner (Figure 3E), and the percentage of apoptotic cells reduced as well (Figure 3F and Supplementary Figure S2). Notably, caspase activities with resveratrol concentrations of ≥15.625 μM were surprisingly lower than mock-infected cells, suggesting that resveratrol could extensively down-regulate apoptosis stimulated outside of VEEV infection.

### 3.5. Molecular Docking Showed Binding of Resveratrol to the VEEV E Protein

**Figure 4:**
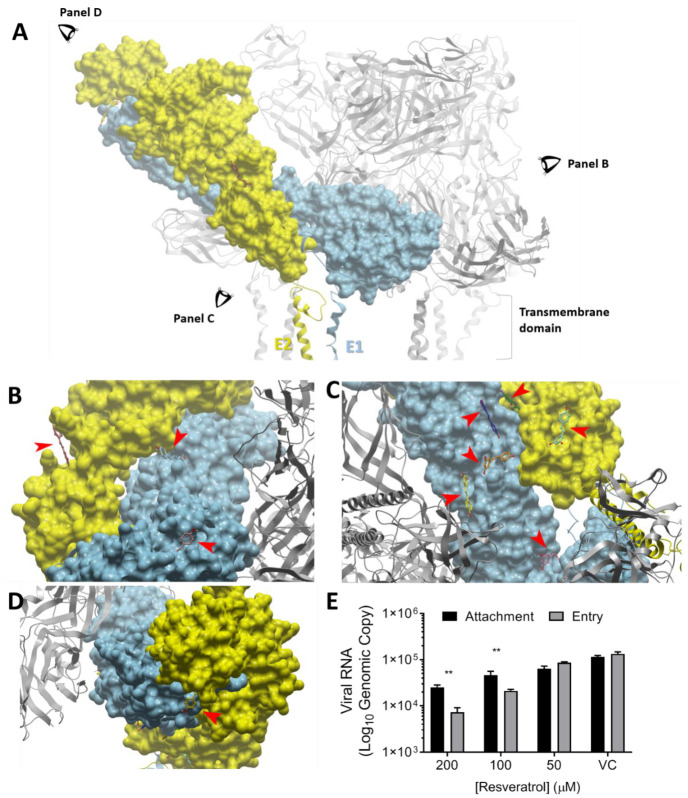
Interaction between resveratrol and VEEV envelope glycoproteins. (**A**) Overview of VEEV E1 and E2 proteins, indicating the viewing angles corresponding to the following panel (**B**–**D**). Red arrows indicate the positions of resveratrol, and E1 and E2 are labeled in indigo and gold, respectively. (**E**) Viral attachment and entry were determined by quantifying the VEEV RNA inside cells at 16 h.p.i. in the presence or absence of resveratrol. ** *p* < 0.01 compared to viral control (VC) by one-way ANOVA.

Polyphenols have been shown to inhibit viral entry [37,38,39,40]. For example, epigallocatechin gallate (EGCG) was documented to impair viral attachment via binding to envelope glycoprotein on Zika virus [41]. As a result, we proposed that resveratrol could also directly interact with the surface glycoproteins of VEEV, which might contribute to resveratrol’s antiviral capability. To explore this hypothesis, possible binding situations in which resveratrol interacts with the VEEV envelope proteins, E1 and E2, were explored using molecular docking. The docking predictions shown in Figure 4 illustrate the most potential docking pockets (lower free energy ΔG, higher binding affinity) within E1 and E2 for resveratrol (arrow-labeled, Figure 4A–D). With further examination of the docking results, we found that two locations might be associated with the anti-VEEV activity: (1) near the fusion loop and (2) near the DI–DIII linker. Both these are found to be important for virus fusion (Supplementary Figure S3) [42], implying that resveratrol might bind to any of these pockets and interfere with the fusion process by forming defective interactions with the fusion loop or the DI–DIII linker. To further refine the docking results via experiments, resveratrol was incubated with VEEV prior to infecting cells (attachment), or alternatively VEEV was allowed to attach the cell surface, followed by the addition of resveratrol (entry). The results indicate that resveratrol curtailed both viral attachment and entry, as shown by the lower levels of viral RNA detected upon resveratrol treatment (Figure 4E). The fact that resveratrol can decrease the amount of viral RNA at attachment and entry stages supports the docking predictions. However, the viral titers at 16 h.p.i. (Supplementary Figure S4A) and the percentage of infected cells (Supplementary Figure S4B) seemed to be equivalent regardless of resveratrol concentrations, suggesting that the interference that occurs between viral glycoprotein and resveratrol is not strong enough to play a long-term decisive role in the anti-VEEV activity of resveratrol.

**Figure 5:**
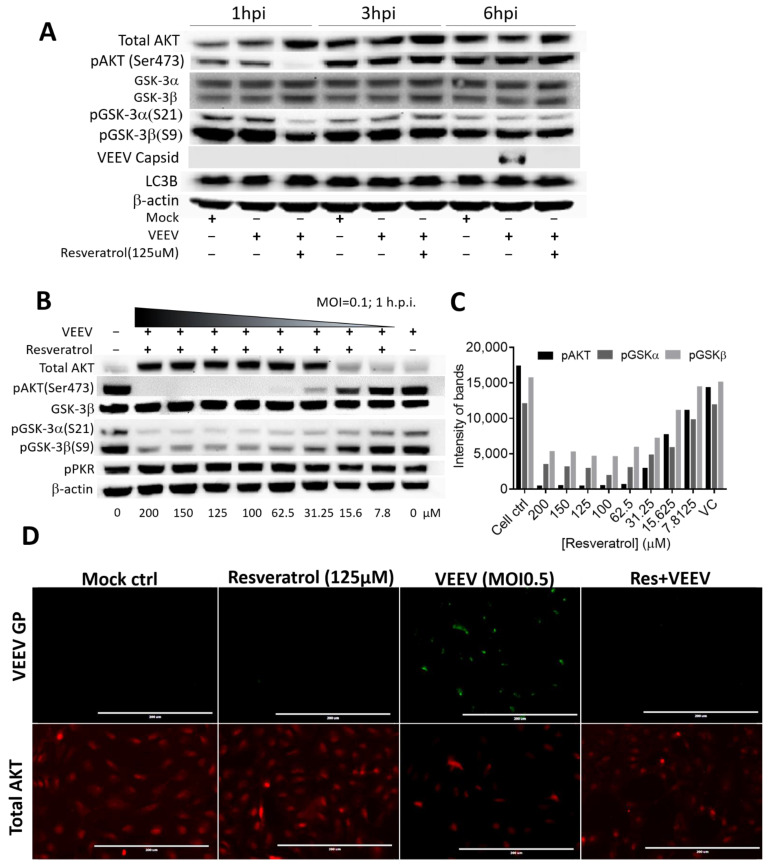
The AKT pathway in VEEV-infected cells after resveratrol treatment. (**A**) Indicated protein level changes in AKT signaling pathways tested at 1, 3, and 6 h.p.i. by Western blot analysis with or without 125 μM of resveratrol along with VEEV-TC-83 infection at an MOI of 0.1. (**B**) The phosphorylation levels of AKT in VEEV-infected cells at 1 h.p.i. after resveratrol treatment at various concentrations and (**C**) the band intensities of AKT, GSK-3α, and GSK-3β were quantified with Quantity One analysis software from Bio-Rad Inc. (**D**) The fluorescent distributions of VEEV glycoprotein (GP) in green and cellular total AKT proteins in red were visualized in mock or VEEV-infected cells at an MOI of 0.5 and 4 h.p.i.

Previous studies have indicated that the AKT pathway is utilized by alphaviruses and could also be regulated by resveratrol [21,22,26,27]. To clarify whether AKT-related signaling may be associated with the anti-VEEV mechanism of action in resveratrol treatment, protein expression and/or phosphorylation levels at 1, 3, and 6 h.p.i. were examined by Western blot analysis. The data revealed that phosphorylation of AKT, GSK-3α, and GSK-3β profoundly reduced after resveratrol treatment at early time points, while the protein levels of total AKT were up-regulated (Figure 5A–C).

To further confirm the Western blot results, total AKT and VEEV glycoprotein (GP) expression in the presence and absence of resveratrol was observed. The images in Figure 5D clearly show that VEEV GP could be detected at 4 h.p.i., whereas the expression of total AKT reduced with resveratrol treatment. The changes in total AKT expression appeared to be consistent with previous results, suggesting that the AKT pathway could play an essential role in the regulation in a resveratrol-treated VEEV infection.

### 3.6. Resveratrol Exhibited Broad-Spectrum Antiviral Activity against Enveloped RNA Viruses

**Figure 6:**
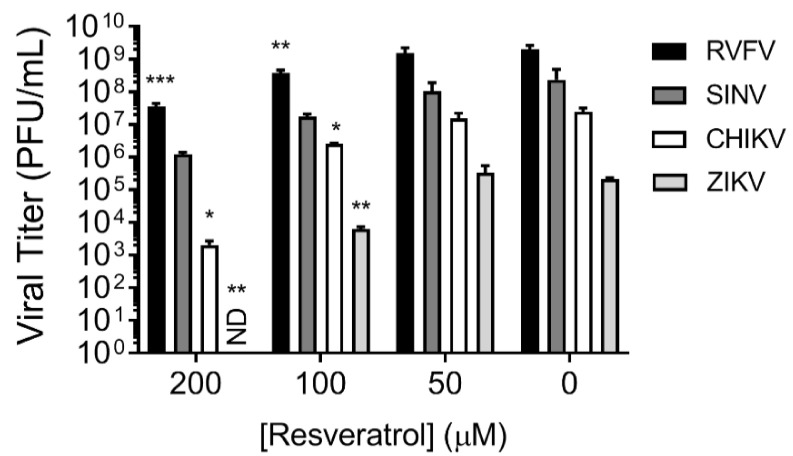
The broad-spectrum antiviral activity exerted by resveratrol. Viral titers of Sindbis virus (SINV), chikungunya virus (CHIKV), Rift valley fever virus (RVFV), and Zika virus (ZIKV) were evaluated at 24 h.p.i. after resveratrol treatment at concentrations from 0 to 200 μM. ND, undetectable. * *p* < 0.05; ** *p* < 0.01; *** *p* < 0.001 compared to no-resveratrol-treated groups by one-way ANOVA.

The literature supports the extensive antiviral activity of resveratrol explored in this paper [43]. To substantiate this point, the broad-spectrum antiviral function of resveratrol was analyzed using other distinct viruses. Viral titers were measured at 24 h.p.i with or without resveratrol treatment at various concentrations by plaque assays. The results (Figure 6) indicate that resveratrol exhibited antiviral activity against Rift Valley fever virus (RVFV; *Phenuiviridae*), Sindbis and chikungunya virus (SINV; CHIKV; *Togaviridae*), as well as Zika virus (ZIKV; *Flaviviridae*), the latter of which has recently been demonstrated in detail [44].

### 3.7. Resveratrol Derivatives Exhibited Comparable Anti-VEEV Activity

**Figure 7:**
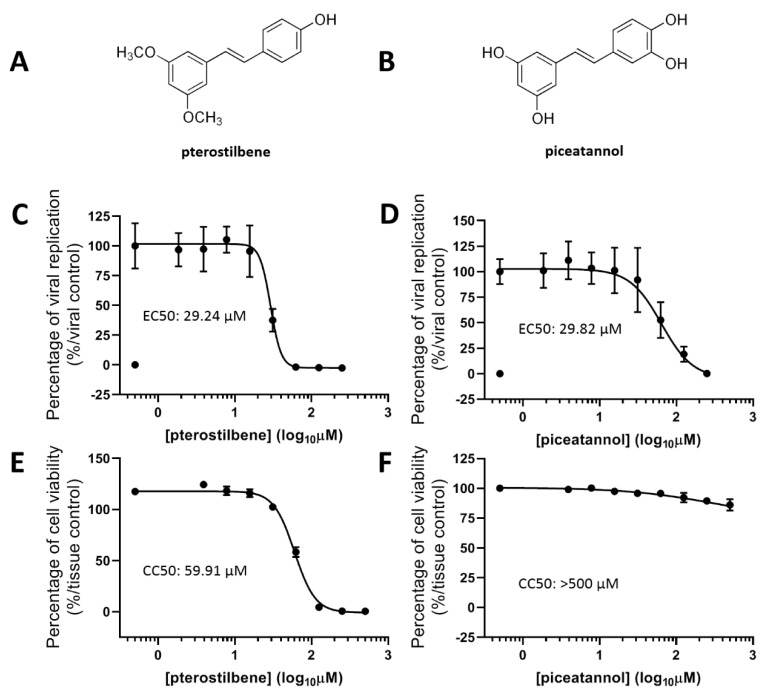
EC50 and CC50 of resveratrol derivatives against VEEV. Pterostilbene (panel **A**) and piceatannol (panel **B**) were used to treat VEEV-TC-83luc (MOI = 0.1)-infected Vero cells at indicated concentrations, followed by measurement of EC50 by viral luciferase activity at 16 h post-infection (**C**,**D**). CC50 values were determined (**E**,**F**) via quantifying cellular ATP levels. The figures were plotted following the normalizing toward tissue or viral control groups.

Finally, to investigate whether resveratrol-like structures might exhibit a different anti-VEEV efficacy, we treated VEEV-infected Vero cells with two resveratrol derivatives, pterostilbene (Figure 7A) and piceatannol (Figure 7B), and evaluated their EC50 and CC50, as described in Figure 1. Our results indicate that both derivatives consistently suppressed VEEV-TC-83 infection and reached comparable EC50 values of 29.24 and 29.82 μM, respectively, compared to that of resveratrol (Figure 7C,D). Surprisingly, pterostilbene exhibited much higher cytotoxicity, whereas piceatannol induced minimal cell death (Figure 7E,F), revealing that the class of resveratrol-related compounds warrants further modification and investigation for the development of anti-VEEV agents.

## 4. Discussion

Resveratrol is a widely used and one of the most understood plant-derived polyphenols. It has been proven to have numerous health-related benefits, including prolonging longevity, maintaining cardiovascular and neurological health, as well as fighting diseases. Despite its long list of benefits, resveratrol has not been explored in the context of veterinary infectious diseases due to the priority placed on human wellness. VEEV infection does threaten the human population; however, humans are infected due to spillover events and are not considered to be the primary host or casualty of the virus. The primary host of VEEV is considered to be equids. In equids, VEEV causes more severe and devastating outcomes when compared to the infection in humans. The current VEEV vaccine provides insufficient protection regardless of TC-83 vaccination and/or C84 as a follow-up booster [45]. Alternative antivirals are urgently needed to control the transmission of VEEV. Our current study demonstrated the potential of resveratrol in combating VEEV infection and elucidated the possible underlying mechanisms of action, including the direct binding of resveratrol to VEEV glycoproteins and the reduction in apoptosis. This information deems resveratrol worthy of future applications and development within veterinary science and neuroprotective research.

The neuroprotective effect provided by resveratrol may play a key role in its anti-VEEV activity. We showed that human glioblastoma cells were protected from cytopathic VEEV infection (Figure 1D,E). The primary damage exhibited on VEEV-infected equines occurs in the brain and spinal cord, manifested by petechia located within the brain and tissues of the meninges as a result of perivascular cuffing and polynuclear monocyte infiltration [46]. In addition to the extensive inflammation initiated by glial cells, there are also subsequently dying neurons exacerbated by the neurodegeneration [47]. In one of our previous publications, we observed that VEEV infection would lead to more necrosis than apoptosis in the same glioblastoma cell line, U87MG, indicating that the neural damage induced by VEEV can be robust and irreversible [48]. On the other hand, the mechanisms of neurological improvement facilitated by resveratrol have been recently depicted in numerous pieces of literature, including enhancement of autophagy, anti-oxidant effectiveness, and direct nerve regeneration [49,50,51]. As a result, the neuroprotectivity of resveratrol is worthy of further investigation as it relates to VEEV infection.

Despite the benefits of resveratrol and preliminary results of its efficacy in vivo (Supplementary Figure S5), it is notable that its utilization has limitations. First, the solubility of resveratrol is relatively low, 0.05 mg/mL, when dissolved in water compared to 87.98 mg/mL in alcohol. Moreover, the stability of resveratrol in mammalian plasma is short. The half-life ranges from 25 to 54 h, depending on the species, despite its high plasma-protein-binding activity [52]. Resveratrol is chemically unstable when exposed to UV light, extreme pH, temperature changes, or intrinsic enzymes of the gastrointestinal tract. This leads to the possible low bioavailability following oral administration [53]. Hence, a better approach to deliver resveratrol directly to the tissues or organs targeted by VEEV needs to be further developed.

An apparent decrease in phosphorylation of AKT and GSK transducers upon VEEV infection was observed in this study, and the expressional changes of the AKT pathway have also been demonstrated to be correlated with the severity of a specific disease [54]. For example, oxidatively damaged neural tissues were ameliorated by resveratrol via enhancing AKT phosphorylation [55]. A clinical study to explore the benefits of resveratrol in diabetic patients revealed that pAKT increased in platelets, resulting in reduced insulin sensitivity [56]. Nevertheless, the detailed mechanisms for pAKT to be up- or down-regulated by resveratrol need to be further clarified.

It is proposed that resveratrol could not only modulate the host survival signaling associated with the AKT pathway but also directly interact with the VEEV glycoproteins. This might lead to the multiple effects of resveratrol against VEEV infection. However, the interaction between the VEEV surface proteins and resveratrol does not seem to be the major mechanism that renders it to be antiviral against VEEV. When resveratrol was co-incubated with VEEV, there was no noticeable decrease in viral titers until 400 μM of resveratrol was utilized (Supplementary Figure S4A). Furthermore, the percentage of infected cells, quantified by the amount of GFP detected, were similar regardless of resveratrol treatment (Supplementary Figure S4B). One possible explanation is that the inhibition of resveratrol toward VEEV attachment and entry could provide partial anti-VEEV effects. So, by the endpoint (16 h.p.i.) of viral titer and GFP+ cell examination, the virus has had the opportunity to replicate numerous times, leading to the inconsequential anti-VEEV activity observed in Supplementary Figure S3.

Taken together, the data illustrate the antiviral effects resveratrol has against VEEV infection, which could be explained as its regulation of the Akt/GSK-3 and apoptotic pathways. The immediate administration of resveratrol slightly impacted anti-VEEV activity; however, subsequent doses allowed for greater anti-VEEV activity. In conclusion, our study suggests that flavonoid-like resveratrol or its derivatives may warrant additional study as prophylactic nutrients or as therapeutic agents for equids upon diagnosis of VEEV, and the AKT pathway may serve as a possible target for anti-VEEV therapeutics.

## Data Availability

Data is contained within the article or supplementary material.

## Supplementary Materials

The following are available online at https://www.mdpi.com/2223-7747/10/2/346/s1: Figure S1: Changes of VEEV replication inhibited by resveratrol at an early phase.; Figure S2: Apoptosis of VEEV-infected cells with resveratrol treatments.; Figure S3: Docking of resveratrol (yellow) in the cryo-EM structure of VEEV E1-E2 glycoprotein.; Figure S4: Direct interaction between resveratrol and VEEV virions.; Figure S5: Resveratrol reduced lethal VEEV infection in mice.
